# Crystallization of Slag Films of CaO-Al_2_O_3_-BaO-CaF_2_-Li_2_O-Based Mold Fluxes for High-Aluminum Steels’ Continuous Casting

**DOI:** 10.3390/ma16051903

**Published:** 2023-02-25

**Authors:** Xiao Long, Shaolei Long, Wenbo Luo, Xiang Li, Changping Tu, Yunhao Na, Jinxin Xu

**Affiliations:** School of Materials and Energy Engineering, Guizhou Institute of Technology, Guiyang 550003, China

**Keywords:** mold flux, CaO-Al_2_O_3_-based, slag film, crystallization

## Abstract

In this study, solidified films of CaO-Al_2_O_3_-BaO-CaF_2_-Li_2_O-based mold fluxes with different contents of Al_2_O_3_ addition were acquired by immersing an improved water-cooled copper probe in bulk molten slags. This probe can obtain films with representative structures. Different slag temperatures and probe immersion times were employed to investigate the crystallization process. The crystals in the solidified films were identified using X-ray diffraction, the morphologies of the crystals were observed using optical microscopy and scanning electron microscopy, and the kinetic conditions, especially the activation energy of devitrified crystallization in glassy slags, were calculated and discussed based on the differential scanning calorimetry. The results indicated that after adding extra Al_2_O_3_, the growing speed and thickness of the solidified films increased, and more time was required for the film thickness to reach a steady state. In addition, fine spinel (MgAl_2_O_4_) precipitated in the films at the early stage of solidification after adding 10 wt% of extra Al_2_O_3_. Together with LiAlO_2_, spinel (MgAl_2_O_4_) acted as nuclei for the precipitation of BaAl_2_O_4_. The apparent activation energy of initial devitrified crystallization decreased from 314.16 KJ/mol (original slag) to 297.32 KJ/mol (5 wt% Al_2_O_3_ added) and 269.46 KJ/mol (10 wt% Al_2_O_3_ added). The crystallization ratio of the films also increased after adding extra Al_2_O_3_.

## 1. Introduction

Mold fluxes are essential materials applied in the continuous casting of steels, which are added to molds during casting and melted to form a slag pool on liquid steel [1,2,3]. Under the action of mold oscillation, molten fluxes in the slag pool flow into the gap between the initial steel shells and water-cooled molds to form the liquid slag film (at the steel shell side) and solid slag film (initially, as a glassy state and then devitrified, at the mold side). The liquid film lubricates the contact between the mold and steel shells for a smooth manufacturing process, whereas the solid film controls the heat flux from the steel shells to the mold for high-quality slab surfaces [4,5,6]. Thus, the structure and properties of the slag films of mold fluxes are of critical importance.

In the continuous casting of high-Al transformation-induced plasticity (TRIP) and twinning-induced plasticity (TWIP) steels, the Al in liquid steel can react with some components in the molten mold flux, especially SiO_2_, dramatically changing the composition of the liquid slag pool and the characteristics of the slag films [7,8,9]. To solve this problem, non-reactive mold fluxes without SiO_2_ and with low content of other active components have been developed; for example, CaO-Al_2_O_3_-based mold fluxes have been proposed as substitutes for conventional CaO-SiO_2_-CaF_2_-based ones. The properties of CaO-Al_2_O_3_-based mold fluxes have been reported, including viscosity–temperature features, solidification processes, heat conduction of solid slag films, and the reaction of molten slags with steels [10,11,12,13,14,15,16]. Numerous CaO-Al_2_O_3_-based mold fluxes have similar viscosity and melting temperature to the conventional CaO-SiO_2_-CaF_2_-based slags. However, their lubricating effect is insufficient during the continuous casting of high-Al steels as sticking or breakout occurs, which significantly limits the industrial applications of CaO-Al_2_O_3_-based mold fluxes. One accepted explanation for the insufficient lubrication is that the overdeveloped slag rims block the slag channel at the meniscus and prevent the liquid slag in pools from flowing into this channel for efficient lubrication [17,18]. In addition, transferring the slag system from CaO-SiO_2_-CaF_2_ to CaO-Al_2_O_3_ changes the structures of solid slag films despite the similar physical properties of the slags. This phenomenon has been reported in our previous work on conventional mold fluxes for peritectic steels and fluorine-free mold fluxes [19,20].

Therefore, it is critical to understand the slag film evolution of CaO-Al_2_O_3_-based mold fluxes. Researchers have attempted to explore the solidification and devitrification of CaO-Al_2_O_3_-based slag films [11,12,13]. However, structures of solid films strongly depend on the cooling conditions. The conventional “water-cooled probe” cannot obtain slag films with representative or similar structures to the films obtained in casting molds [21,22]. Thus, there are very limited reports on the structural evolution, such as thickness growth speed, devitrification features, the roughness of surfaces in contact with molds, and the porosity of the solid slag films of CaO-Al_2_O_3_-based mold fluxes under cooling conditions similar to those in molds.

Crystallization in solid slag films (containing devitrified and solidified crystals) plays a key role in controlling the heat flux by reflecting and scattering infrared rays from steel shells [23]. In addition, crystallization in glassy films potentially increases the roughness of CaO-Al_2_O_3_-based film surfaces in contact with the mold wall (although this is insignificant in CaO-SiO_2_-CaF_2_-based slag films) [19]. Thus, this work aims to reveal the crystallization, especially the devitrified crystallization features, of typical CaO-Al_2_O_3_-based slag films similar in cooling conditions to films in molds and their possible influence on the casting process.

To obtain slag films with representative structures and study their evolution process, an optimized water-cooled copper probe was adopted in this work. It has been proven to yield solid slag films with structures similar to those obtained in molds. The structure, characteristics, and advantages of this optimized water-cooled probe can be found in previous studies [21,22].

## 2. Materials and Methods

A typical CaO-Al_2_O_3_-based mold flux was selected as the basis of this study (No. 1 slag in Table 1). To adjust the physical properties of the slag, fusion agents (BaO, CaF_2_, LiF, Li_2_O, B_2_O_3_, and MgO) were added. As endogenous inclusions containing Al_2_O_3_ in liquid steel can be absorbed by molten mold fluxes and increase the actual Al_2_O_3_ content in slag films, 5 wt% and 10 wt% of extra Al_2_O_3_ were added to the typical CaO-Al_2_O_3_-based mold fluxes (as No. 2 and No. 3, respectively) to reveal the effect of Al_2_O_3_ absorption on the crystallization process. The slags for the experiments were prepared using analytical-grade reagents (CaCO_3_, Al_2_O_3_, BaCO_3_, CaF_2_, LiF, Li_2_CO_3_, B_2_O_3_, and MgO). The reagents were loaded into a high-purity graphite crucible and pre-melted in a resistance furnace at 1300 °C, which were then poured and quenched in a water-cooled copper plate.

For each solidification experiment, 300 g of pre-melted slag lump was loaded into a high-purity graphite crucible with an inner diameter of 60 mm and melted in a resistance furnace. The optimized water-cooled copper probe with a large width–thickness ratio (20 mm wide, 15 mm tall, and 6.35 mm thick) was immersed in liquid slag to acquire a solid slag film. To study the effect of slag temperature on film crystallization, three different slag temperatures (1300 °C, 1350 °C, and 1400 °C) were used. In addition, different probe immersion times (7, 15, 30, 60, and 90 s) were adopted to investigate the evolution processes.

### Measurements and Analysis

The thickness of the slag films was measured using a point micrometer. For each film sample acquired on the wide face of the probe, six measurements near the film center were adopted. The average value of these six measurements was taken as the film thickness. The crystallization process was investigated by inspecting the cross-sections of the films using optical microscopy and scanning electron microscopy (SEM, FEI Company, Nova Nanosem 450, Hillsboro, OR, USA) with energy-dispersive spectroscopy (EDS). Cross-section samples for SEM measurements were prepared by mounting solid films into resin, polishing with alumina suspensions, and coating with Au. The crystals in the films were identified through X-ray diffraction (XRD, Cu K_α_, Rigaku Miniflex 600, Tokyo, Japan) after grinding the solid films into powder. The precipitation ratio of various crystals in the slag films was measured using EDS on a cross-section area of 4 mm^2^ for each sample. 

The kinetic conditions of the film devitrification were investigated using differential scanning calorimetry (DSC, NETZSCH STA 449 F3, Selb, Germany). Samples for DSC measurements were prepared by grinding pre-melted slag (quenched in a glassy state) into a powder with a diameter of less than 74 μm. Four different heating rates (5, 15, 25, and 35 °C/min) were applied for DSC measurements. The crystallization activation energy of glassy slag can be calculated using the Kissinger equation [24], as shown in Equation (1).

(1) $$ln\frac{\beta }{T_{p}^{2}}=A-\frac{E_{K}}{RT_{P}}$$

where *β* is the heating rate, *T_P_* is the temperature of the first peak on the DSC heating curve (the temperature of the obvious initial crystallization), *A* is a constant related to the system characteristics, *E_K_* is the apparent activation energy of devitrified crystallization, and *R* is the gas constant.

## 3. Results and Discussion

### 3.1. Evolution of Thickness and Devitrification of Slag Films

**Figure 1:**
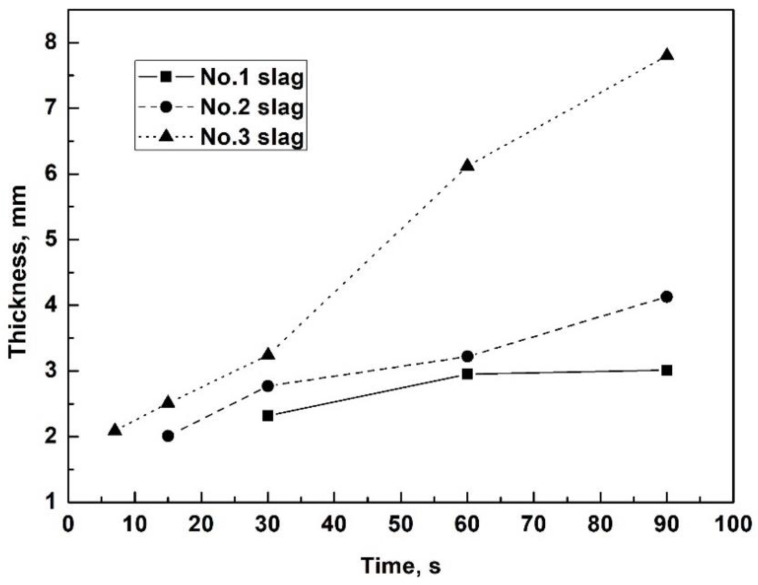
Average thickness of slag films solidified in slag bulk at a temperature of 1350 °C.

The thicknesses of the obtained slag films are listed in Table 2. When the solidification time was short, especially 7 s, some films could not be obtained because they did not tightly adhere to the water-cooled probe, or the adhered film was incomplete. As shown in Figure 1, when the slag temperature was 1350 °C, the thickness of No. 1 slag films reached a steady state after approximately 60 s solidification. As the Al_2_O_3_ content increased from No. 1 slag to No. 3 slag, the thickness of the solid films increased dramatically and an extended time was required to reach a steady thickness. With a decrease in slag temperature, the film thickness increased significantly, indicating that the temperature fluctuation of the liquid slag could cause unevenness of the solidified slag films, especially for No. 3 slag.

**Figure 2:**
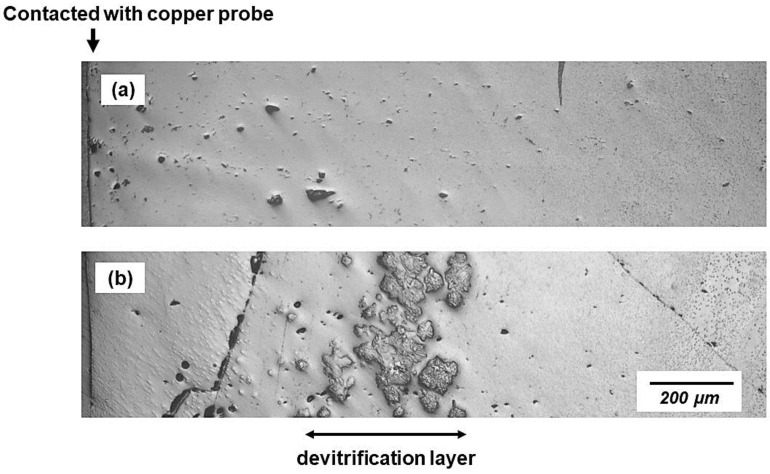
Optical images of cross-sections of solid slag films with different solidification times, (**a**) 15 s, (**b**) 30 s of No. 1 slag (bulk temperature 1300 °C).

**Figure 3:**
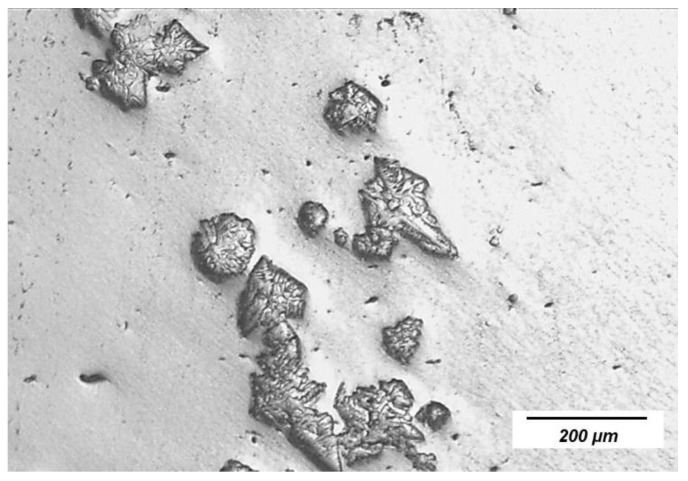
Optical image of crystals dispersed in glassy film (slag bulk temperature 1300 °C).

Figure 2 shows a typical devitrification layer in a solid slag film. In addition to the labeled devitrified crystallization layer, fine crystals started to precipitate on the probe side (Figure 2b). Obvious cracks with healing signs and isolated flat hole chains were evident, consistent with the observation in F-free slag films in previous works [25]. The formation and healing of these cracked surfaces could cause heat flux fluctuations, especially during the early stage of solidification. This was partly due to the shrinkage of the nearby devitrified crystallization. Notably, some devitrified crystals were dispersed in the glassy films, as shown in Figure 3; thus, it is difficult to measure the thickness of the devitrification layer.

### 3.2. Crystals in Slag Films

**Figure 4:**
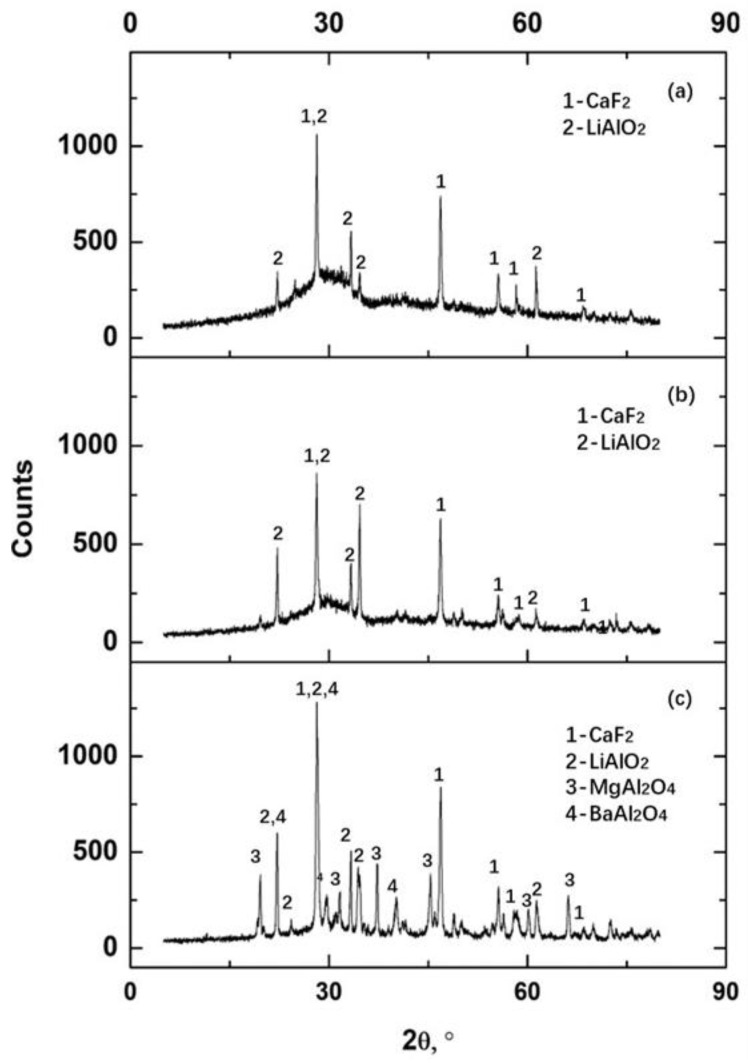
Typical XRD patterns of slag film powders: (**a**) No. 1 slag; (**b**) No. 2 slag; (**c**) No. 3 slag. Bulk slag temperature for film solidification: 1350, and solidification time: 30 s.

The XRD patterns of typical slag film powders are shown in Figure 4 and the major crystal phases are summarized in Table 3. Calcium fluoride, lithium aluminate, and barium aluminate were the major crystals in most of the films. In the films with an extra 10% alumina (No. 3 slag), spinel (MgAl_2_O_4_) with a high melting temperature was detected.

**Figure 5:**
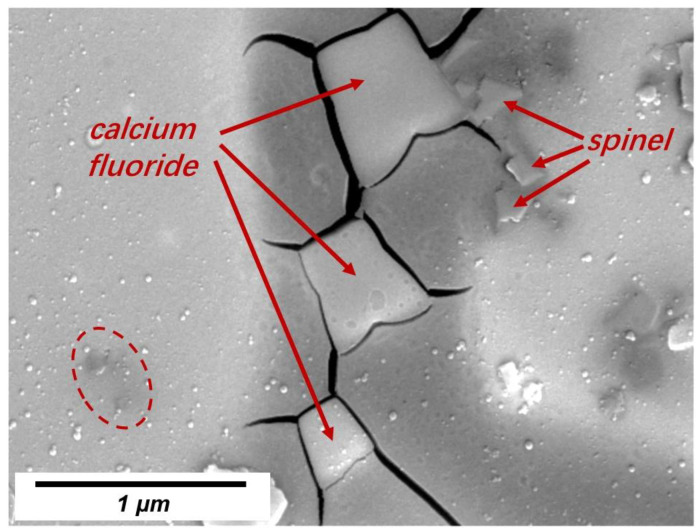
Backscattered SEM image of typical morphology of the devitrified crystals in the No. 3 slag film solidified in molten slag at 1300 °C.

**Figure 6:**
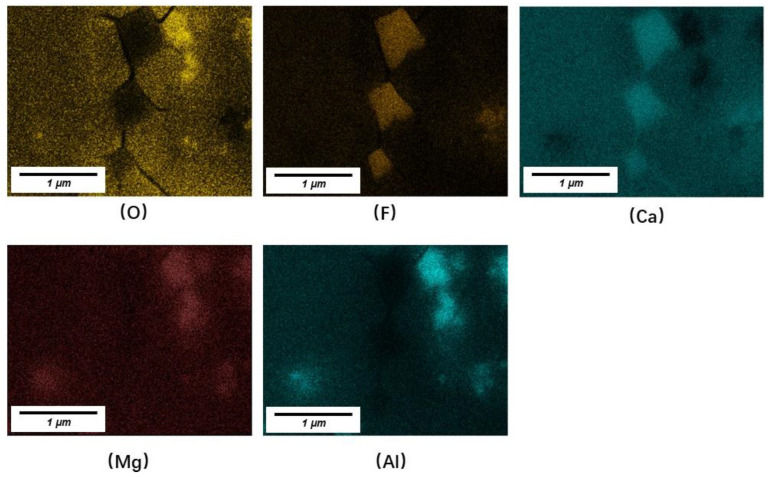
EDS mapping of CaF_2_ and spinel (MgAl_2_O_4_) crystals.

To verify the existence of spinel, the cross-section of No. 3 slag film (devitrified parts) was inspected using SEM-EDS. Typical backscattered SEM images and the corresponding elemental maps are shown in Figure 5 and Figure 6, respectively. Based on the previous X-ray diffraction results presented in Table 3 (to identify the kind of precipitated crystals in films) and the corresponding elemental maps shown in Figure 6, precipitated calcium fluoride (containing Ca and F) and spinel (containing Mg, Al and O) were identified. The dark area around the calcium fluoride indicated that the crystals did not solidify in the liquid slag but precipitated in the glassy film. In addition to the labeled spinel, very fine spinel crystals were observed in the film (circle in Figure 5). The EDS map (Figure 6) revealed that Mg, Al, and O were concentrated in the circled area. The fine spinel particles precipitated at the solidification front, especially in the liquid slag film, could increase the apparent viscosity of the film, which was unfavorable for lubrication. This increased viscosity was also one of the reasons for the fast-growing film thickness.

In this study, only 2 wt% MgO was added and fine spinel crystals were detected in the slag, indicating that the absorption of Al_2_O_3_ from liquid steel or MgO from raw materials of slags and corroded refractory should be restricted.

It should be noted that massive cracks were observed around nearly all calcium fluoride crystals. Further research is required to investigate the effects of cracks on the thermal resistance of the solid slag films.

**Figure 7:**
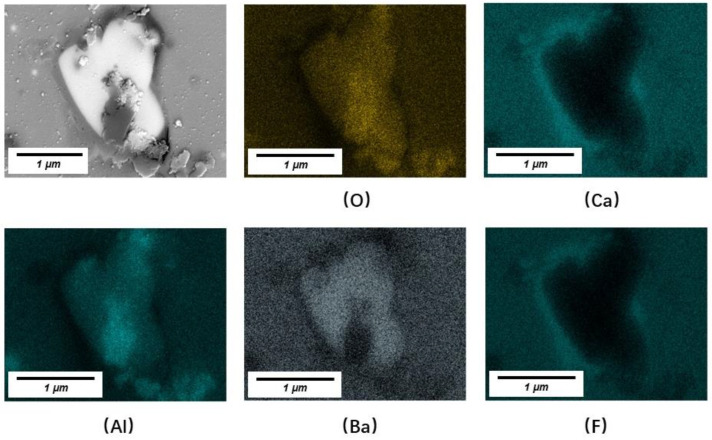
Backscattered SEM image and EDS area scanning of LiAlO_2_ and LiAlO_2_ cores for the precipitation of BaAl_2_O_4_ in the film solidified in molten slag at 1300 °C.

Table 3 reveals the precipitation sequence of crystals. For No. 1 slag, CaF_2_ and LiAlO_2_ precipitated first followed by BaAl_2_O_4_ when the probe immersion time exceeded 60 s. For No. 3 slag, spinels precipitated at a very early stage with CaF_2_ and LiAlO_2_ followed by BaAl_2_O_4_. Because barium ions are larger than the other ions, their diffusion for crystallization requires more energy and time. This is one of the reasons that barium oxide can partially replace calcium oxide to decrease the crystallization capacity of mold fluxes under certain circumstances, especially mold fluxes for alloy steel casting to dissolve various non-metallic inclusions. Figure 7 shows BaAl_2_O_4_ precipitated on the LiAlO_2_ core (only Al and O were detected at this core using EDS point analysis). As Li cannot be detected using EDS, a distribution map of Li is not presented.

### 3.3. Kinetic Conditions of Crystallization

**Figure 8:**
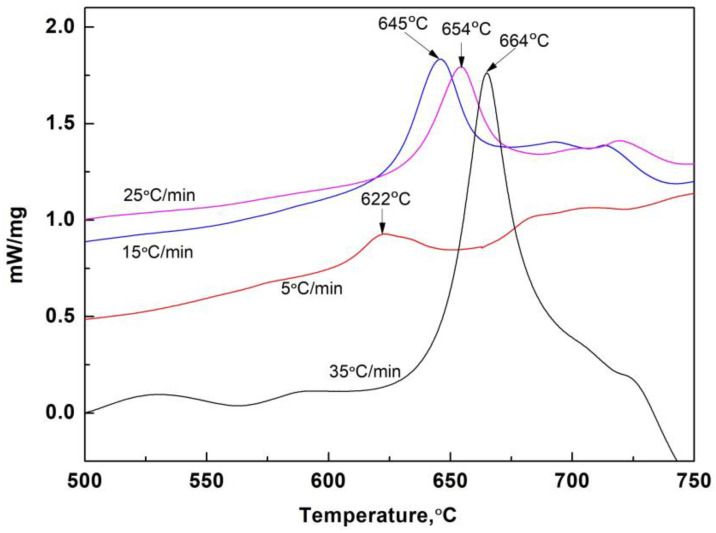
DSC curves of quenched glassy powder of No. 1 slag with different heating rates.

**Figure 9:**
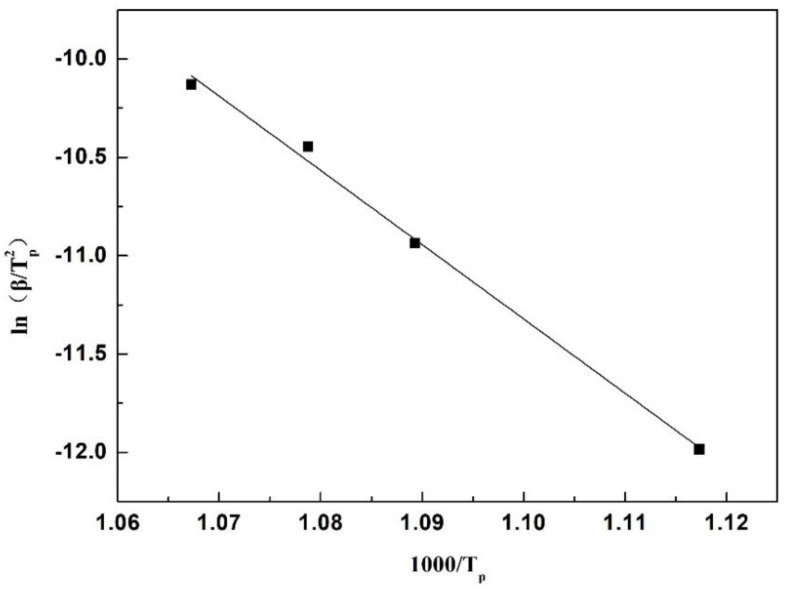
Relationship between *ln*(*β/Tp*^2^) and 1*/T_P_* of No. 1 slag.

Typical DSC curves of the No. 1 mold flux powder (glassy state) at different heating rates are shown in Figure 8. With an increase in heating rates from 5 °C/min to 35 °C/min, the initial temperature of apparent devitrification increased from 622 °C to 664 °C. Based on the Kissinger relationship in Equation (1), the plot between *ln*(*β/Tp^2^*) and *1/T_P_* is shown in Figure 9. For No. 1 slag, the calculated apparent activation energy of devitrified crystallization was 314.16 KJ/mol. Based on the same methods, the calculated apparent activation energies of devitrification for slags 2 and 3 were 297.32 and 269.46 KJ/mol, respectively, indicating that the devitrification capacity of the slag increased after adding Al_2_O_3_. The increase in devitrification capacity provided more nuclei for crystallization, which partially contributed to the increased crystal ratio of films, which is discussed later.

The DSC results also indicate that the heating rate can affect the softening temperature zone of glassy films. In Figure 8, when the heating rate reached 35 °C/min and the temperature exceeded 700 °C, the slag began to absorb heat and became soft or melted. This feature was not observed at lower heating rates. Slow heating rates provided better kinetic conditions and allowed sufficient time for LiAlO_2_ and CaF_2_ to precipitate in the glassy film. The consumption of Li, Ca, and F ions in the nearby glass matrix significantly decreased the Li_2_O and CaF_2_ fusion agents in the remaining glass. Consequently, the softening or melting temperatures of the nearby glass matrix increased. The softening of solid slag films could decrease the surface roughness of solid films in contact with the water-cooled copper wall, which was also observed in our previous study [19]. This phenomenon could affect the capacity of heat transfer control of solid slag films during solidification; in addition, it could be utilized in the design of the property of the solid slag film for high-Al steels. Detailed research on this topic is ongoing.

**Figure 10:**
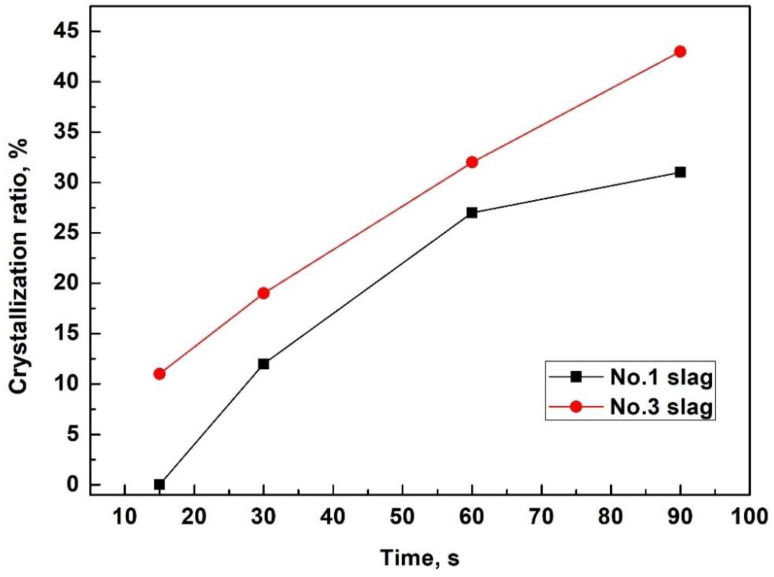
Crystallization ratio evolution of typical slag films solidified in molten slag at 1400 °C.

**Figure 11:**
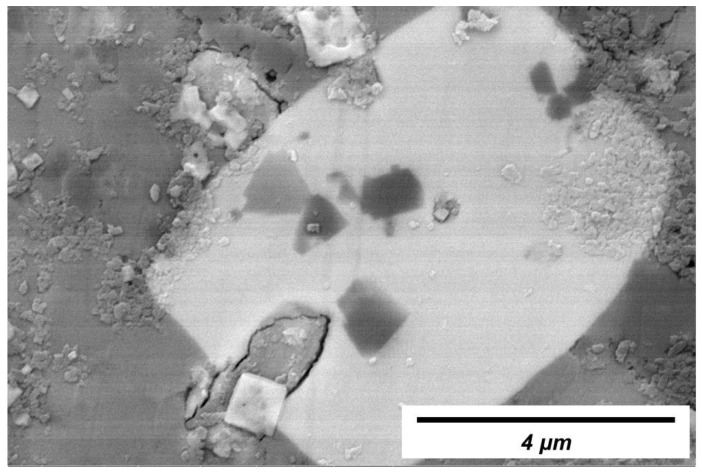
A typical crystal (BaAl_2_O_4_) precipitated using initial crystals as nuclei (backscattered image, the No. 3 slag film solidified in molten slag at 1300 °C).

The area ratio of a specific crystal on the cross-section was determined as the crystal ratio. The measured crystal ratios in the typical slag films are listed in Table 4 and Figure 10. A comparison of the No. 1 and No. 3 slag films indicated that extra Al_2_O_3_ increased the crystal ratio and crystallization capacity of the films. Consistent with the previous discussion on Figure 7, during the crystallization of slag films, the initial precipitated LiAlO_2_ and MgAl_2_O_4_ usually acted as nuclei for the further precipitation of BaAl_2_O_4_ (also see Figure 11). Thus, the low apparent activation energy of the devitrified crystallization of No. 3 slag could provide more nuclei at a very early stage of film solidification, promoting the precipitation of crystals in the glassy film and liquid slag with a relatively high viscosity near the solidification front.

## 4. Conclusions

The crystallization process of slag films of typical mold fluxes for casting high-Al steels was studied in this work, and the following conclusions can be drawn:

(1) After adding extra Al_2_O_3_, the growing speed and thickness of the slag films increased significantly, especially for films with 10 wt% Al_2_O_3_ addition, and more time was required to reach a steady state of film thickness.
(2) LiAlO_2_, CaF_2_, and BaAl_2_O_4_ were the major crystals precipitated in slag films of No. 1 and No. 2. BaAl_2_O_4_ usually precipitated and used initial crystal particles as their nuclei. Spinel (MgAl_2_O_4_) precipitated in the films when an extra 10 wt% of Al_2_O_3_ was added. The results suggest that the MgO in the slag or the absorption of Al_2_O_3_ from liquid steels should be controlled.
(3) For the glassy state of No. 1 slag, when the heating rate reached 35 °C/min during devitrification, the softening temperature decreased to approximately 700 °C. Lower heating rates were associated with higher softening temperatures.
(4) The apparent activation energy of initial devitrification crystallization for the original slag (No. 1) was 314.16 KJ/mol. After adding Al_2_O_3_, this activation energy decreased to 297.32 KJ/mol and 269.46 KJ/mol for 5 wt% and 10 wt% Al_2_O_3_ addition, respectively. The crystallization ratio of the slag films also increased after adding extra Al_2_O_3_.

## Figures and Tables

**Table 1:** Composition of mold fluxes (wt%).

No.	CaO	Al_2_O_3_	BaO	F	Li_2_O	B_2_O_3_	MgO
1	24	32	15	10	6	4.5	2
2	24	37	15	10	6	4.5	2
3	24	42	15	10	6	4.5	2

(All components are represented as oxides, except for F).

**Table 2:** Average thickness of slag films with different solidification times and slag bulk temperatures.

Solidification Time, s	No. 1 Slag			No. 2 Slag			No. 3 Slag		
	Bulk Temperature of Molten Slag, °C								
	1400	1350	1300	1400	1350	1300	1400	1350	1300
	Average Film Thickness, mm								
7	-	-	-	-	-	-	1.61	2.09	3.35
15	-	-	2.54	1.67	2.01	3.29	1.88	2.51	4.37
30	1.73	2.32	2.99	1.89	2.77	3.97	1.98	3.24	5.60
60	2.23	2.95	4.45	2.68	3.22	4.33	2.62	6.12	-
90	2.45	3.01	5.56	2.71	4.13	6.21	2.97	7.80	9.17

**Table 3:** Major crystal phases in films with different solidification times and slag bulk temperatures.

Solidification Time, s	No. 1 Slag			No. 2 Slag			No. 3 Slag		
	Bulk Temperature of Molten Slag, °C								
	1400	1350	1300	1400	1350	1300	1400	1350	1300
	Crystal Phase								
7	-	-	-	-	-	-	G	G	AC
15	-	-	B	G	AB	AB	BC	ABC	ABC
30	AB	AB	AB	AB	AB	ABD	ABC	ABCD	ABCD
60	ABD	ABD	ABD	ABD	ABD	ABD	ABCD	ABCD	-
90	ABD	ABD	ABD	ABD	ABCD	ABD	ABCD	ABCD	ABCD

(A-CaF_2_, B-LiAlO_2_ C-MgAl_2_O_4_; D-BaAl_2_O_4_; G-totally glassy state).

**Table 4:** Typical crystal ratios of films solidified at different times (%).

Slag No.	Solidified Time, s	CaF_2_	Spinel (MgAl_2_O_4_)	LiAlO_2_	BaAl_2_O_4_	Crystal Ratio of Film
No. 1	30	2	0	10	0	12
	60	8	0	13	6	27
	90	9	0	15	7	31
No. 3	7	0	0	0	0	0
	15	0	3	8	0	11
	30	5	3	11	0	19
	60	7	4	14	7	32
	90	12	4	16	11	43

The film was solidified in molten slag at 1400 °C.

## Data Availability

Not applicable.
